# Emergency Department presenting complaints associated with high mortality and the need for intensive care

**DOI:** 10.1186/1757-7241-20-S2-P16

**Published:** 2012-04-16

**Authors:** Jakob Lundager Forberg, Charlotte Barfod

**Affiliations:** 1 Department of Emergency Medicine, Hillerød Hospital, Denmark; 2 Department of Anaesthesiology, Hillerød Hospital, Denmark

## Background

Mortality associated with specific diagnosis is well described. However, the mortality and adverse outcome related to the presenting complaint in the Emergency Department (ED) is less well studied. Identifying ED patients’ presentations associated with high risk for adverse outcome could help us improve early treatment and optimise prioritization in the ED.

## Methods

During six months all patients admitted to the ED at Hillerød Hospital were prospectively registered in the Acute Admission Database. The chief complaint was registered during triage according to the Hillerød Acute Process Triage protocol and categorized into 41 presenting complaints. Intensive care unit (ICU) admissions and mortality data were retrieved from the hospital administrative system (OPUS Arbejdsplads, CSC) and merged with triage data. The majority of patients in a Danish ED are referred for admission by their general practitioner (GP) or by an out-of-hours GP service. The ED receives patients with any complaint, except for direct referrals within gynaecology, urology, paediatrics (medicine) and major trauma.

## Results

6335 unique patient contacts were registered of which 380 (5.9%) patients did not have a presenting complaint registered. Thus 5955 patients were included in the study. The overall ICU admission rate was 1.6% and mortality within one month after admission was 4.2%. The ten presenting complaints with highest one month mortality were: Altered level of consciousness (15.6%), vomiting blood (10.4%), diarrhoea (10.2%), dyspnoea (9%), cough (8.9%), aphasia (8.7%), symptoms of hip fracture (8%), abnormal laboratory results (7.7%), vomiting (7.4%) and unilateral extremity weakness (6.3%). The ten presenting complaints associated with the highest risk of admission to ICU were: hypertension (6.3%), altered level of consciousness (5.6%), dyspnoea (5%), vomiting blood (3.9%), diarrhoea (3.4%), blood in stool/melaena (3 %), seizure (2.9%) and drug poisoning/overdose (2.9%), abnormal laboratory results (2.8%) and hypo/hyperglycaemia (2.3%).

## Conclusion

Identifying presenting complaints associated with high mortality or increased risk of admission to ICU are important in order to optimise resources and the early treatment in the ED. Presenting complaint with a traditional high focus and resource allocation, e.g. chest pain and trauma, are not associated with the highest mortalities.

